# Sensory Labor as Un/knowing in a Waste Composition Study: Identifying a Chain of Translation

**DOI:** 10.1177/03063127251357625

**Published:** 2025-07-23

**Authors:** Taru Lehtokunnas, Niina Uusitalo, Ulla-Maija Sutinen, Alma Onali

**Affiliations:** Faculty of Social Sciences, Tampere University, Tampere, Finland

**Keywords:** multisensory ethnography, translation, waste composition study, sensory labor, waste policy

## Abstract

To keep track of the types and proportions of waste produced in society, various waste composition studies are carried out around the world. Through ethnographic fieldwork, this article examines how statistical knowledge about waste was produced in the context of a waste composition study organized by a Finnish waste management company. We follow a chain of actions involving sensory labor to show how waste was translated into numerical knowledge. By shedding light on the sensory labor of producing knowledge on waste, we contribute to social scientific waste research, especially by illustrating how the interplay between senses and waste—as matter that can evoke strong sensations—challenges the possibilities of translating waste into numerical knowledge. We also show how these difficulties are, in turn, managed to create accurate data for the purposes of waste policies. In addition, our study contributes more widely to the public discussion on waste policies by highlighting the role of sensory labor as crucial for the operation of the circular economy.

A truck dumps a thousand kilograms of mixed waste on the concrete yard. A forklift arrives, spreading the bags evenly across the plane. We stand there, in sweltering summer heat, wearing white plastic overalls, protective glasses and masks, and a couple of layers of gloves, with shovels and pitchforks in hand. Seagulls circle the grounds of the waste-management site. After the forklift leaves, we shovel waste bags and loose dirt—mattresses, dolls, rotting apples, cat litter, yesterday’s dinner—into a waste container, stopping when we reach 100 kilograms. This amounts to a high heap above the container.

After collecting the sample, we push it into a large tent that resembles a hangar. There are three tables inside the tent: one for biowaste and two for mixed waste. The tables are covered with sieves. A circle of plastic buckets of different sizes is arrayed around the tables. Altogether, there are 36 categories for mixed waste items. We place the first bags on top of the sieves and open the bags with a knife, revealing their warm, smelly contents, and start to pick apart and categorize every single little piece of matter found in the bag. Bag after bag, gag after gag, we dissect and categorize hundreds of kilos of excess matter. The work is laborious, numbing, visceral, disgusting. A couple of weeks later, all of this sensory labor is a neat number in a statistical report.

Waste composition analyses, such as the one we describe here, are conducted to produce statistical data on the relative proportions of different materials in waste streams and to gain knowledge of the sorting habits of populations. Usually, these analyses are applied to household or community waste. Evaluating both the accuracy of sorting practices in households and the composition and volumes of waste they produce are central means of creating more effective waste policies in the context of the circular economy (Liikanen et al., 2016; Silvennoinen & Nisonen, 2023). The circular economy is a model that aims to ‘close the loops’ by making materials circulate through practices such as reducing, reusing, and recycling (Ghisellini et al., 2016), as opposed to the current linear economic model of take–make–use–dispose. The statistical information concerning different waste fractions found in mixed waste is publicly shared through media reports and information and marketing campaigns of waste management companies. Thus, this data has a central role in educating consumers to improve their consumption and sorting habits, in the process enacting ‘good citizenship’ (see Ibáñez Martín & de Laet, 2018). Moreover, the results of waste sorting studies make it possible to evaluate how effectively the national and EU-level circular economy goals concerning recycling rates are accomplished.

Our research team volunteered to assist in a waste composition study conducted by a regional waste management company in Finland, as it presented an excellent opportunity to get close to waste flows and the knowledge production processes that guide current waste policies. The company had its own reasons for producing data, but our interest was in the sensory and bodily processes related to knowing waste. We approached the study from the perspective of multisensory ethnography (Pink, 2015; Vannini, 2024), a growing field of enquiry that promotes the sensory and bodily aspects of knowledge production.

In general, informal and alternative waste regimes, such as through the practices of waste-pickers (Coletto & Bisschop, 2017; Lehtonen & Pyyhtinen, 2020; Reno, 2009; Thieme, 2013) or the socio-materiality of urban waste landscapes (Fiore, 2024; Hird, 2012), have been extensively studied, whereas the official, technologically driven waste management process remains understudied from a practice perspective, apart from some exceptions, such as Nagle’s (2013) ethnographic account on how New York City’s Department of Sanitation operates to manage waste. Various waste composition analysis methods have been examined and compared (e.g. Dahlén & Lagerkvist, 2008; Karppinen et al., 2021), but the concrete processes of the bodily and sensory labor of conducting a waste composition study are rarely explicitly described.

In this study, we examine the problematics of knowing and unknowing waste in the context of waste composition studies (Alexander & O’Hare, 2023). To do this, we draw from the concept of translation (Haraway, 2016; Latour, 1987, 1999) and connect it with discussion on knowledge production as a multisensory process (Alcayna-Stevens, 2016; Fiore, 2024; Pink, 2015; Vannini, 2024). More specifically, we utilize the concept of sensory labor (Spackman & Lahne, 2019), which refers to the productive aspects of senses. In the case of the waste composition study, our senses were harnessed to serve the waste economy by producing knowledge for the purposes of waste governance and policy. We study how the translation of waste into statistical data happens through practices of categorizing waste through sensory labor, and how these categorizations often entail multiple imperfections and compromises (see e.g. Woolgar & Neyland, 2013).

Our study contributes to social scientific waste studies (see e.g. Alexander & O’Hare, 2023; Douglas, 1966; Hawkins, 2006; Hird, 2012; O’Brien, 1999; Thompson, 1979) by illustrating the specific features of sensory labor as a form of knowledge production when handling waste. This article also contributes to discussions concerning waste policies in the context of the circular economy by showing the difficulties of generating statistical knowledge on waste, and more generally, by showing how the accuracy of the practices of categorizing waste is sometimes hindered by the interplay between senses and waste. Based on our analysis, we argue that to create more sustainable circular economy policies, instead of technocratic attempts to make waste completely manageable (Abrahamsson, 2023; Alexander & O’Hare, 2023; Genovese & Pansera, 2021), it would be crucial to better acknowledge the role of sensory labor both in the production of knowledge and economic value.

It is important to note that sensory labor with waste has different methods and different objectives depending on the economic, political, and cultural context (Morais et al., 2022). For instance, waste pickers’ co-operatives in Buenos Aires use creative practices to classify and recycle waste and have also achieved key improvements in their working and living conditions, due to their progressive inclusion within municipal waste management policies (Carenzo, 2020). Pickers of menstrual waste in India also organize labor and work to destigmatize waste in general (Vaughn, 2020). In contrast, in the Global North, doing sensory labor with waste can be a political choice to enact alternative, sustainable lifestyles, as in the case of some food dumpster divers in Finland (Lehtonen & Pyyhtinen, 2020). Furthermore, while waste pickers do sensory labor to extract value from and revalue waste materials (Carenzo, 2020; Gregson et al., 2010; Morais et al., 2022), the formal waste composition study has no such interest. Our work as waste composition analysts was to categorize without valuation or hierarchical judgement. We aimed to sort as many items as possible, as accurately as possible, to contribute to the information needs of the official waste management regime operating in the Nordic, highly technologized and high-income context. Thus, even though all examples deal with sensory labor with waste, they take part in very different spheres of global circular economy.

In the following, we first describe this study’s way of knowing and discuss our methods of generating the data and conducting the analysis. After this, we establish the theoretical basis for our study and discuss previous work on the various dimensions of knowing and not knowing waste. We also elaborate on the literature on the body, the senses and bodily ways of knowing in the context of producing scientific facts. Furthermore, we link this discussion with the concepts of translation, sensory labor and categorization, that will be applied in our analysis. After laying our theoretical groundwork, we explore our main empirical insights, which centre on three phases of translating waste through sensory labor and categorization: collecting samples, categorizing waste and weighing and quantifying the waste. We conclude by discussing how our work contributes to social scientific waste studies and broader discussions on waste policies in the context of the circular economy.

## On our method of knowing

This article draws on ethnographic fieldwork conducted within a two-week waste composition study organized by a Finnish waste management company in autumn 2023. Five members of our research group participated in the day-to-day work of the waste composition study for one to two weeks. While working as members of the waste composition study workforce, we used our researcher bodies and senses as the primary ‘instruments’ for conducting ethnographic fieldwork (Hammersley & Atkinson, 1995). Our data consist of individual field notes written by the authors of this article, transcriptions of discussions recorded on our joint car rides home from the waste centre, photographs and video material recorded with an action camera. The data gathering continued after the actual waste composition study ended. After the active fieldwork, we had meetings in which we discussed and shared our thoughts and experiences. As part of one meeting, we engaged in creative writing tasks based on our individual experiences. The structure of these discussions resembles the feminist memory-work research method (e.g., Onyx & Small, 2001; Valtonen et al., 2020). The meetings produced further collective conceptualizations of different aspects of the fieldwork. The data analysis followed an iterative process, cycling repeatedly back and forth from the data to our analysis and interpretation (Tracy, 2013). The visual data (photos and videos) functioned as complementary data and memory aids.

Our research can be thought of as a study within a study. In our roles as social scientists, we took part in an official waste composition study that would have been carried out without our presence. The coexistence of two different studies taking place at the same time and our dual roles as ethnographers and waste composition study workers was sometimes challenging. Participating in the waste composition study was physically demanding work, and our bodies were often exhausted after the workday. While we wanted to pay extra attention to our senses, under such conditions, this was sometimes an overwhelming challenge (we discuss this further in the analysis). On a more practical note, due to the rather fast pace of the work and the fact that we had to wear protective gear, there were few opportunities to write field notes during the workdays, and even taking pictures and videos required extra effort.

In addition to balancing the roles of ethnographers and waste composition study workers, our roles as ‘ordinary consumers’ who are typically separated from the materiality of waste made its presence felt in many ways. Quite rapidly, the waste composition study revealed that our bodies reacted to waste in very different ways: Some were more sensitive to smells to the point of gagging, and others reacted more viscerally to worms. We also had a considerable amount of prior knowledge about waste; it was assumed that we could identify most waste materials based on our previous experience as members of consumer society. As Harris (2020, p. 4) notes, sensing is not something innate, cognitive, or individual but rather cultivated through social, bodily and material practices. Indeed, we found that we had plenty of knowledge about what different materials feel and look like, and how, for instance, certain food items crumble at a touch. Sometimes, we also felt like intruders into people’s private lives as we went through their waste piece by piece—a practice that is not typically socially accepted.

## The social scientific approach to waste and knowledge production

This article is informed by social scientific waste studies (see e.g. Douglas, 1966; Hawkins, 2006; O’Brien, 1999; Thompson, 1979), especially more recent discussions concerning the dynamics of knowing and not knowing waste (Alexander & O’Hare, 2023; Hird, 2012). Social scientific waste researchers have noted that the efficient waste infrastructures and waste management practices currently in place (especially in the Global North) allow people to be ignorant about the waste they produce after discarding, as it is taken care of by someone else (Hawkins, 2006), although the level of awareness and ignorance varies according to sorting practices and infrastructures in different contexts. However, aims to enhance recycling and the circular economy currently dominate the logic of waste policy, which shapes everyday practices of managing waste. We are expected to wash, store, sort and recycle waste. As we already discussed in the introduction, waste composition studies are conducted to study the accuracy of these sorting practices by measuring the proportions of different waste fractions in mixed waste. This kind of measurement produces knowledge about waste and recycling rates, but problems of unknowing are also always present, both in the uncertainty of practices of measuring waste and because the use of ‘objective’ statistical data as a tool in waste policy potentially creates ignorance about other possible ways of living with and knowing waste, such as the sensory and embodied forms of knowing (see Alexander & O’Hare, 2023; Ibáñez Martín & de Laet, 2018; Poovey, 1998).

In discussions concerning ignorance and not knowing, especially in the field of science and technology studies (STS), scholars have noted that, in the context of scientific and technical innovation, knowing and not knowing are always produced simultaneously, because the consequences of the produced knowledge and its applications are always unclear (Böschen et al., 2006; Ravetz, 1987). The dynamic relation between knowing and not knowing has also been discussed specifically in relation to waste. Alexander and O’Hare (2023) distinguish five different categories of technologies involved in ‘unknowing’ waste: spatial, temporal, epistemological, calculative and rhetorical. First, spatial technologies are those pertinent to geographical issues (e.g. shipping waste to other countries), hierarchical borders (e.g. local, national or global), scale (a perspective or category related to waste that often displaces others) and separation (e.g. separating waste treatment facilities from people’s living environments). Second, temporal issues refer to practices of placing waste to the past or future, which can mean, for example, technologies for storing nuclear waste. Third, there are epistemic issues connected with secrecy and denial when dealing with polluting, toxic and dangerous wastes. Fourth, calculative technologies concern measurement, such as in the creation of figures on the global production or movement of different waste streams. Finally, the category of the rhetorical is linked to all the aforementioned categories and refers to certain rhetorical techniques of framing waste that simultaneously produce certain ways of knowing, such as calling waste a ‘resource’.

In this article, we focus on epistemic and calculative issues when discussing the waste composition study as a specific technique for knowing waste. However, when studying the knowing/unknowing of waste, we extend the focus from the secrecy and denial that relates to the practices of treating toxic waste. By exploring the composition study of regular household waste, we illustrate how the dynamics of knowing and unknowing relate to practices of examining waste matter that is smelly, intimate, soiled, and sometimes so disgusting that you would not want to touch it, see it, or even know about its’ existence. This also relates to the calculative dimensions of knowing—we show how the practices of measuring waste are possible regardless of this paradox of producing knowledge on stuff you do not want to know about.

## The sensory labor of categorizing and translating waste

The past few decades have seen growing interest in the body and its materiality in the social sciences, especially within feminist scholarship (e.g. Alaimo & Hekman, 2008; Barad, 2007; Haraway, 2016; Mol, 2002). Examining the body and its materiality (e.g. diseases, senses, affects) is crucial for understanding issues such as ‘lived experience, corporeal practice and biological substance’ (Alaimo & Hekman, 2008, p. 4). We can understand the study of the senses and the ‘sensory turn’ (see e.g., Allen-Collinson & Owton, 2015; Hayes-Conroy & Hayes-Conroy, 2008; Pink, 2015) as part of a wider interest in the body and its materiality in the social sciences.

Alongside many studies in STS that have examined the messy realities of laboratories that produce ‘scientific facts’ (Garforth, 2012; Knorr-Cetina, 1999; Latour & Woolgar, 1979), there is a large body of research into sensory ways of knowing. In addition to studies that examine vision in the context of scientific practices (e.g. Haraway, 2016; Henderson, 1999; Jenks, 1995), researchers have, for example, examined how hearing is an integral aspect of examination (Mody, 2005), how the nose is trained to detect specific smells (Latour, 2004; Muniesa & Trébuchet-Breitwiller, 2010; Spackman, 2020) and explored tasting as a scientific practice (Butler, 2018; S. E. Tracy, 2018). In healthcare contexts, researchers have studied the importance of tactile knowing (Lammer, 2007; Pink et al., 2014). Drawing on a conception of the subjects and objects of scientific practice as constantly ‘in the making’, Alcayna-Stevens (2016) articulates the bodily practices of field scientists habituating themselves with the natural environment of bonobos, shedding light on how versatile the body and all its senses must be to conduct this type of animal behavior study—even though this embodiment is often left out of the final reports, as if it never played a role.

Following this line of research, we examine the process of translation (Haraway, 2016; Latour, 1987, 1999) of the anonymous, smelly and disgusting waste masses into different kinds of information, namely statistical and ethnographic knowledge, through the concept of sensory labor. Spackman and Lahne (2019) write about sensory labor in the context of tasting and discuss how mouths and noses create economic value in industrialized food systems by producing knowledge about collective preferences of consumers related to the taste of foodstuffs. Sensory perception through smelling and tasting thus makes sensing bodies part of economic systems of production. In the case of the waste composition study, our sensory labor of categorizing waste objects through touch, smell and visual classification became part of knowledge production concerning the sorting practices of households in the context of the circular economy transition. This knowledge is crucial in the context of the circular economy, as the possibilities of circulating materials into new cycles of production depends on the sorting practices of consumers.

From the viewpoint of the chain translating waste into statistical knowledge, categorization of waste through sensory labor was crucial, turning the anonymous waste masses into governable and calculable objects. Each phase in the chain of translation thus entailed multiple practices of categorization. This categorization, however, was in no way straightforward or flawless, as our analysis shows. As Bowker and Star (1999) write, categories are often invisible, as is the work that produces them, yet they have a lot of power and real-life consequences. Moreover, real-world classifications hardly ever meet the theoretical requirements of classificatory systems. Woolgar and Neyland (2013) illustrate the tensions between real-world categorization and requirements of classificatory systems through an ethnographic study in a municipal waste management center. They show how the waste classification system within the center could be maintained only through constant repair work by the employees, and how households tended to generate their own waste classification schemes due to unclear and ambiguous waste sorting instructions.

In addition, waste has some specific features that, in its interplay with senses, may create some specific classification problems. As waste scholars have noted, our relations with waste are marked with visceral registers, such as disgust (Hawkins, 2001; Hawkins & Muecke, 2003; Kinnunen, 2021). Waste evokes (sometimes overwhelming) sensory reactions and effects that relate to its abjectual (Kristeva, 1982) nature: To maintain order and our own subjectivity, we usually want to separate ourselves from the deformed and indeterminable, such as a waste bag’s worm-covered, smelly, slimy, and warm contents. Our analysis of the sensory labor of categorizing waste highlights how the sensory labor conducted with waste requires constant efforts of coping and stretching one’s sensory tolerance. As our analysis illustrates, the stretching of tolerance also relates to the paradoxes of knowing/unknowing discussed in the previous section.

## Identifying the chain of translation of waste

In this section, we discuss how our bodies were trained to conduct the sensory labor (Spackman & Lahne, 2019) of categorizing waste in order to translate the matter into statistical knowledge. We identified three phases in the chain of translation in which we took part: 1) collecting samples, 2) determining categories and 3) weighing and quantifying the waste. In our analysis, we show how we applied and combined our senses to create knowledge within the chain of translation. Training the body to carry out the sensory labor of categorizing waste was not a straightforward process. Therefore, we also illuminate how the interplay between the senses and waste, as well as the setting itself, introduced barriers to the categorization of waste in different phases of translation. There were other phases in the chain of translation that we glimpsed or heard about, but in which we were not able to actively take part. We return to these phases in our conclusion.

### Collecting samples: Translating loads of waste into as-reliable-as-possible samples

The first phase in the chain of translation we identified was sample collection. In this phase, a load of waste collected from different neighbourhoods by a waste truck needed to be translated into as-reliable-as-possible samples from which the average composition of household waste could be concluded. The shift from a load of waste into a sample was the first transformation (see Latour, 1999) the waste went through during the composition study.

**Figure 1.**
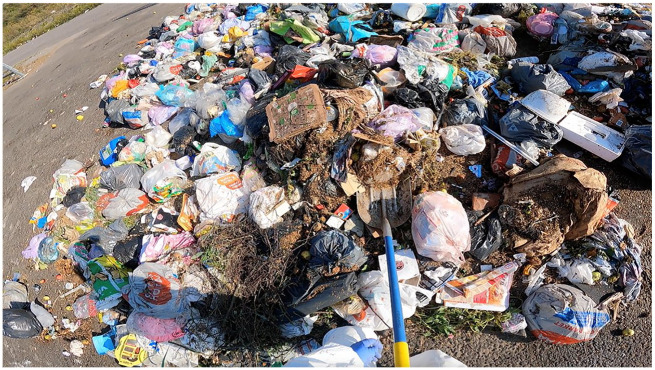
A 100-kg sample of waste was gathered by shovelling waste and handpicking waste bags and items into a container.

First, the waste truck deposited a 1,000-kilogram load of household waste outside the tent in which the sorting part of the composition study was conducted. This load was spread evenly across the ground so that we were able to identify and collect individual waste bags. First, large and heavy items, such as tires, mattresses, wooden furniture, metal items and carpets, were taken from the waste load and weighed separately, to avoid skewing the distribution of different materials. After this, we had to collect a 100-kilogram sample in a sample container, which was later brought inside the sorting tent. The container was first placed on a pallet truck equipped with a scale to weigh the sample. After that, the collection of the sample began. The waste bags had to be picked out from all around the area to ensure that the sample included bags from different households. We also had to be careful to collect more fine-grained waste particles, such as cat litter or soil, that were not in the waste bags, using a shovel to move them to the sample. Usually, there were two to four people collecting the sample, while others continued sorting previous samples in the tent or tidied up the area surrounding the sorting tables (Figure 1).

To conduct the labor of translating the waste load into a sample that could later be sorted and measured, we used multiple senses. We had to use our vision to collect waste bags of different colors and sizes for the sample: Waste bags of the same color may have come from the same households. We were also often able to superficially assess what the bags contained by looking inside or assessing the shape of the bag. For example, we had to avoid collecting too many bags of garden apples, as apples are quite heavy. In addition to using vision when collecting the bags, the feeling of heaviness or lightness was crucial in collecting the sample. There were many ways to feel the waste bags: When we collected the sample, we walked among the bags and occasionally stepped on them, stuck them with our shovels or kicked them, which enabled an embodied assessment of the type of stuff the bag potentially contained.

In this sense, the sensory labor (Spackman & Lahne, 2019) of categorizing the items for the sample was conducted through co-configuration of the senses—vision operated together with tactile sensations of the heaviness or lightness of bags and other embodied assessments of the contents of each waste bag. Thus, in different phases during the chain of translation, different senses formed a system of perception that enabled the categorization of waste (both in the sense of recognizing waste objects from each other and choosing suitable objects for the sample).

Although the categorization of objects suitable for the sample happened through the co-configuration of vision and tactile sensing, there were also some general technical tips, provided by employees of the waste management company. We were instructed to divide the waste load into different sections by creating a visual ‘grid’ in our heads, and then to make sure that we took something from each section of the grid. When the sample started to come close to 100 kilograms, we began to yell out the scale numbers to each other, and this also influenced whether heavier or lighter bags were picked up from the load and added. Moreover, when collecting large items from the load, we negotiated over issues such as where to place the different items and whether certain items were large or heavy enough to be taken from the load.

While we were guided through the procedure of collecting the sample, the process varied each time, due to the heterogeneity of the waste material. This complicated the process of categorizing objects suitable for the sample, and also made the sensory labor more difficult, as we had to always re-orient the system of sensory perception to recognize different objects. The contents of each waste load were different, and even if the loads were equally heavy, they looked (and sometimes smelled) different. As was noted several times by multiple people during the composition study, handpicking waste for a 100-kg sample introduces the possibility of aberration.

I still think that creating representative samples is super hard with this type of stuff. If you just try to take regular waste bags, it isn’t representative. Because, for example, some textiles may be thrown out in their own bags if you clean out a wardrobe or something. So, there is probably quite a lot of variation in the results because of that. (Car ride discussion, 18 August 2023)

As the composition study proceeded through the weeks, our embodied, sensory knowledge about waste also started to affect our practices of collecting the sample. We came to understand that it was good to collect both heavy and light waste bags for the sample, but at the same time, we learned that the heavier bags that both looked and smelled damp—and felt that way when poked with the shovel—often contained something disgusting, such as diapers or rotten organic residue. Moreover, the colors of the waste bags started to give some information about their contents; waste bags from bathrooms were often pink, purple, or blue, while waste bags from kitchens were often reused plastic bags with grocery store logos. One of us recalled how picking up certain waste bags started to cause visceral effects of disgust and repulsion and how this created an internal negotiation: ‘Now that I touched it, I need to take it.’ This had to be done to avoid allowing these bodily reactions to ‘disturb’ the sample collection.

Thus, the embodied knowledge acquired through sensory labor sometimes created a temptation to avoid certain waste bags when collecting the sample, which could have shaped the chain of translation (see also Hardahl et al., 2019; Latour, 2004), and certainly created tensions. Embodied and other ways of knowing waste did not necessarily make the knowledge production process more reliable—sometimes quite the opposite (see also Alexander & O’Hare, 2023). The temptation not to know was thus constantly present in the knowledge production process: We did not always want to know what the disgusting, damp and smelly waste bags contained, especially after we had gained more experience about the potential contents of these bags—and still this knowledge was crucial for the study.

### Determining categories: Translating masses into as-categorizable-as possible objects

After the sample was collected, the main part of the day-to-day work in the waste composition study was assigning a category to each piece of waste. This work of translating waste masses into as-categorizable-as possible objects constituted the second phase and transformation of waste in the chain of translation. It should be noted that ‘mixed waste’ is itself a product of categorization, a process that flattens the multiplicity of heterogeneous matter into one neat, symbolic, manageable category. Our task was to pick apart the category of ‘mixed waste’ and to classify it again into its parts, to *know* what the category included. This highlighted the material aspect of waste.

**Figure 2.**
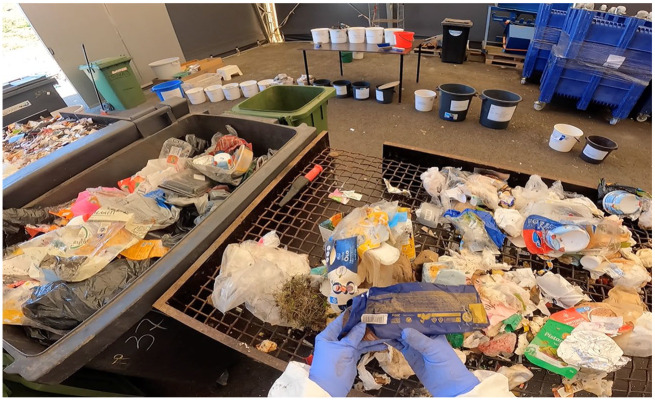
The waste was divided into categories on sorting tables with metal grates on top. Each category of waste had its own plastic bucket or container around the sorting tables.

The sorting procedure started by placing a couple of waste bags on sorting tables from the sample container. The bags were opened either with knives or by hand. We had to be careful not to stick our hands directly into the bags since they may have contained, for example, sharp objects. Instead, we were advised to empty out the bag on the table or create a large hole in the bag to see its contents fully. The sorting tables had grates through which smaller particles fell to the bottom of the table so that they could be weighed separately after the whole 100-kilo sample was sorted (Figure 2).

Once the trash bag was opened on the table, each item from the bag had to be categorized before it could be removed from the table. The established waste categories were mostly based on the material components, such as metal, paper, and plastic. The waste bags that we opened on the table were usually crammed with plastic packaging from food products, paper towels, biowaste, maggots, ash, cat litter, indeterminable fluid, cotton pads, and many other items tangled up with each other. Formerly distinct objects had turned into anonymous masses in the bags. Our job was to make sense of this mess by categorizing the anonymous masses into objects that were, at least to some extent, recognizable (see also Lehtokunnas & Pyyhtinen, 2023). This meant that if, for example, a plastic package of minced meat was full of cotton pads and maggots, the pads had to be identified as separate objects and removed from the package (the maggots stayed with the matter to which they were stuck).

The categorization of waste masses into identifiable objects required that we used multiple senses to conduct the labor. For certain materials, there were tricks and cues for checking the material. For instance, to detect whether a yoghurt lid was made of plastic or metal, we were advised to use the ‘wrinkle test’; metal will stay wrinkly, while plastic straightens. Training the body to differentiate materials required knowing and observing the qualities of the materials. Determining whether an opaque package still had something in it was done by shaking and listening to the item as well as trying to estimate the weight of the product.

I noticed what I was doing with my body. I shake, I sway, I squeeze, I rattle, I crumple, I listen, I look attentively. I shake deodorant containers and other cosmetic containers. By shaking a container, one can determine surprisingly well if there is something inside or if it’s just the package. Probably somewhere from one’s spine arises an estimation. … The lids of yoghurt packages need to be crumpled to know whether they are plastic or metal. Medical packages need to be rattled and listened to to know whether they are empty. Multiple senses are needed to separate the materials. (Extract from Ulla-Maija’s field diary)

Thus, it was often not enough to visually identify a certain object; categorizing objects was done by creating a system of sensory perception through the simultaneous use of vision, touch, shaking and listening, and other actions. Mixed waste sometimes also included biowaste, which required specific sensory techniques from the sorters. Sometimes, biowaste needed to be smushed between one’s fingers before one could tell the difference between, for instance, a paper towel and a potato. This had to be done since, as all waste items get mixed and start to rot in the waste pickup process, they become less easily identifiable. However, even as we learned these multiple bodily strategies for translating masses into knowledge categories, a certain element of unknowing (see Alexander & O’Hare, 2023) was always present. For example, it was impossible to tell whether a piece of cling film came from product packaging or from a cling film roll that someone used at home. In this sense, sensory knowledge was not always enough to ensure completely reliable categorizations, but all the objects had to be classified regardless. Even though one would be able to use the senses to identify an object, its’ origins affect how it should be categorized, and these origins, in turn, may be impossible to detect in some cases.

A further element obstructing the fluent categorization of waste masses was the need to wear protective gear. We had to protect ourselves both from the sensory qualities of waste and from the bacteria that waste contains. We used gloves (three sets for some of us), which created a physical barrier to the sense of touch and covered the tips of our fingers, which in everyday settings we use to sense and detect different materials. We also had to wear face masks and protective goggles to protect our respiratory organs and eyes from the waste. This safety equipment made it possible to complete the work, by keeping the smells and textures of the waste at bay, but it made us feel sweaty and uncomfortable, steamy protective goggles obstructed sight, and gloved fingers were not able to sense the waste directly.

The waste still took a heavy toll on our senses. The general invasive smell of waste in the sorting tent made us not want to breathe through our noses; at the same time, breathing through the mouth was not always easier, as one can also smell through the mouth. There were also certain types of waste, such as feces and blood, that we tried not to look at too closely, to avoid bodily reactions. Dealing with the repulsiveness of waste meant that we had to use our senses but limit them, developing a type of bodily discipline (Muniesa & Trébuchet-Breitwiller, 2010); we had to be brave enough to swing, squeeze, and poke different waste items to recognize them, but careful to not examine certain items too closely, to avoid gagging. Due to sheer disgust, some things may not have been sorted correctly.

Identifying correct categories for the waste was a constant process of probing and interpreting individual fragments and items. Sorters habitually consulted with each other to identify and classify waste fragments into the correct (or agreed-upon) fractions. ‘Which bin have you put X in?’ was frequently asked. Asking for guidance from one another was often a quick way of knowing and involved a calibration of senses between sorters (Harris, 2020). Thus, single acts of translating the waste mass into recognizable objects were often the results of joint negotiation rather than precise efforts either to identify objects using our own individual senses, or to study the rules of the composition study in the instructions we were given by the waste management company (see Woolgar & Neyland, 2013). Despite the written instructions and discussions with colleagues, we came to learn that individual sorters occasionally interpreted the waste fractions differently; sometimes, they corrected their sorting, and at other times, they let these interpretations become part of the margin of error. As previous studies on waste categorization practices have shown, waste classification schemes are often ambiguous, and as a result people create their own schemes and interpretations (Woolgar & Neyland, 2013). Thus, the sensory labor of categorizing waste was in many ways based on shared practices as well as individual interpretations. The sorted waste objects were not fixed, but rather viscous—they were constituted by multiple different agencies and relations between objects, people, and places (Persaud et al., 2019). And in some cases, a correct categorization would have required more knowledge about the origins of the object.

### Weighing and quantifying the waste: Translating waste categories into as-accurate-as-possible numbers

Finally, when the 100 kilograms of mixed waste had been sorted through and only tiny ‘smushes’ of waste were left on the table, the weighing process began. Weighing and quantifying the waste was the third phase in the chain of translation that we identified as part of the composition study process and through which the sorted waste was transformed into numerical data.

To weigh the categorized waste, one worker sat in front of a laptop and opened an Excel form. A scale whose screen showed the numbers was positioned next to the person in charge of recording all the data in the Excel form. Other workers started to prep for the weighing; some were weighing the waste in bigger containers with a pallet jack scale, and some were already combining the contents of buckets carrying the same categories of waste. The weighing often started with the contents of the bigger containers, such as plastic packaging, and these numbers were yelled out to the scale ‘manager’. This person started to call out the categories in their numerical order (each fraction had a number), shouting, for instance, ‘and then glass packages and glass’. Others brought the completed buckets and containers and positioned them on the scales. Typically, buckets were queued up in front of the scales, waiting for their turn to be weighed. Once the buckets were weighed, their contents were emptied into bigger containers. After this, the buckets were brought back to their correct places around the sorting table. All the carefully categorized waste fractions were once again treated as mixed waste.

After all the waste fractions had been weighed in their designated containers, the waste smush that had fallen through the grates of the sorting tables needed to be weighed. In this sense, the smush, too, needed to be categorized at the end of the sorting process of each sample, but this categorization was different. The smush was first carefully examined to identify the different proportions of waste that it contains (e.g. coffee grounds and small plastic particles), and overall composition percentages were roughly estimated. This evaluation was conducted through reliance on vision and touch. We, however, often had to rely more on visual evaluation than touch, since the different materials that the smush contained were often thoroughly mixed with each other and it was difficult to separate them.

This process also involved some negotiation, as different sorters had different ideas about the correct percentages. Differences related, for example, to the difficulties of visually evaluating the weight of different proportions of waste. Sometimes, for example, the smush seemed to contain a lot of plastic and only a small proportion of coffee grounds, but wet coffee grounds are much heavier than plastic, and because of this, a larger percentage was given to biowaste. In these cases, the system of sensory perception needed to be oriented to visually evaluate weight, and some of the sorters (especially the employees of the waste management centre) were more trained to do this. This hindered the consistency of the practices of categorization through sensory labor on some occasions.

**Figure 3.**
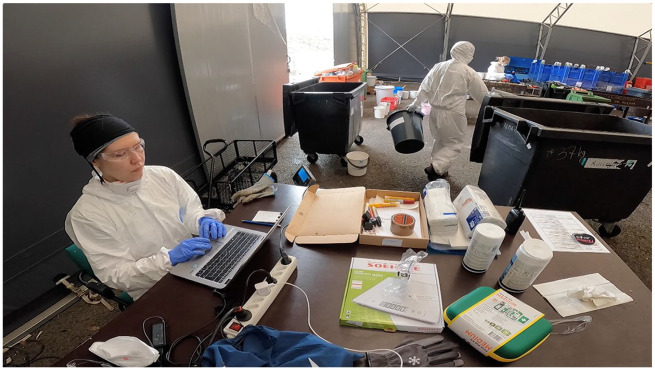
One researcher documented the weight of each category in an Excel sheet, while others brought containers to the weighing station and emptied them into the mixed waste.

After evaluating and recording the percentages of different proportions, the mixture was then swept into its own container and weighed. Weights for all of the waste containers and buckets when empty were written on the sides, and this information was also included in the Excel sheet. Excel constantly calculated the overall amount of waste weighed, and in the end, someone checked whether this number adequately matched the sample’s original 100 kilograms (Figure 3).

Although the weighing required lifting and moving the containers containing the sorted waste, it did not require dealing with the waste as closely as the other phases of conducting the study. However, after several weigh-ins, one could get an overall idea of the typical distribution of waste into different categories, and exceptions to this were noticeable. For instance, one day, the textile waste buckets were almost overflowing compared to earlier weigh-ins, and an extra note about this was added to the Excel form. In this case, the knowledge gained during the same phase of translation with earlier waste loads contributed to the accuracy of the weighing and helped us recognize major exceptions to ‘the usual’. Altogether, in a sense, the inaccuracy of the knowledge produced through the sensory labor of categorizing waste (during the earlier phases) was tempered through the process of weighing, which was a more direct and distinct way of producing knowledge and ‘hard’ quantitative data for the composition study.

However, the production of this quantitative data was not straightforward or flawless. The process allowed for errors at the weighing stage, as the scale did not work accurately at times, and exhaustion sometimes caused the numbers at the weighing station to be heard incorrectly. Still, weight was the most essential factor in the composition study, as the end result was reported in kilograms. While plastic and cardboard containers took up most of the space in the sorting containers, the most weight came from biowaste. The final weigh-in never amounted to 100 kg, which means that part of the waste escaped during the sorting process. Some of this was undoubtedly liquids or small particles that escaped from the sorting stations. Moreover, and related to weight being the most essential factor in the study, some of us noticed a change in our thinking after spending a few days categorizing waste:

Now that I have spent four days in the field, I have started to better understand the logic of the composition study. So far, I have mainly focused on identifying different objects, but today I realized that the weight of different materials is more relevant than focusing on whether single objects are categorized correctly. For example, it is not very relevant whether one packed lollipop is categorized correctly, as it is so light that the scale does not even recognize it. (Taru’s field diary, 17 August 2023)

As we gradually came to grasp the ‘big picture’ of the composition study, our ways of knowing waste were shifted. At the beginning of the study, we focused mostly on the recognition of individual objects, such as lollipops, chicken legs or diapers. Towards the end of the study, we became more aware that, in this context, knowing waste required understanding the different material qualities of waste, especially the weight and composition of particular materials. Only the weight of each categorized waste fraction was recorded in Excel—the accurate recognition of individual objects did not matter at this point, as long as the categorizations were done more or less correctly. This also was apparent in our discussions with the employees of the waste company. One worker, for example, said that she does not find waste disgusting, as she mostly just thinks about materials when sorting. After each 100 kg sample was sorted, the experience of gagging, touching worms, and fighting the stench of waste was translated into a neat Excel file that, in principle, provided valid data for comparison.

The inaccuracies of the sensory labor during the first two phases of translation were managed through the practice of weighing at the end of sorting each sample. In this sense, although the whole chain of translation was based on categorizations of individual objects, in the end, masses were more relevant than individual objects from the viewpoint of knowledge production. Further, categorizing the proportions of waste in Excel produced a certain technique of unknowing (see Alexander & O’Hare, 2023) in which all the tensions, flaws and contradictions in the process of categorizing waste through sensory labor became invisible.

## Concluding discussion

On the last day of the composition study, there were not many sorters at work, so we only had to deal with a couple of samples. After that, we started the process of cleaning the equipment. Buckets, knives, and shovels were taken outside the sorting tent, close to another building with a water supply. The equipment was washed with a hose; strong water pressure made the buckets jump and fly out of the washing place. The buckets did not have to be perfectly cleaned, though; no one would use them for purposes other than waste composition studies in the future. They were left in the sorting tent to dry, and after they had dried, someone would pile them up. The larger waste containers, the floor of the sorting facility, and the outside area where the trucks unloaded their contents, were left to be tidied up and washed by bigger machinery. Finally, we put the computers, papers, and scales in a cart and left the sorting site. We also finally got rid of the protective gear; overalls, gloves and glasses were binned, and shoes were to be left in the back space of the changing room. Our ‘waste sorting laboratory’ was disassembled, and so was our very special and peculiar bodily connection to waste. Waste turned once again into a black box from which we would now be able to maintain a certain distance in our everyday lives.

As mentioned earlier, the phases of translation we have explicated in detail were not the only stages involved in the composition study. In fact, we were only part of the translation process for a short (yet crucial) period. A large part of the sorting work was already done before the composition study started; garbage truck routes were planned and scheduled so that there would be enough loads of waste from predefined locations for each day of the study. During the study period, other workers at the waste facility spread out the waste loads and picked up the waste after the samples were collected and sorted. Furthermore, after the composition study period, the figures were calculated, and results from different samples were combined. After that, the knowledge was transformed into communications that we later read in the local newspaper and press releases. The fact that we did not take part in these processes is a clear limitation of our study, but at the same time, our primary aim in this article was to analyze the sensory labor of translation.

The sensory labor of categorizing waste created a chain of translation that altered the ontological quality of the waste (Latour, 1987, 1999). During the composition study, the waste gradually underwent different transformations: It turned from general mass into a sample, from a sample into categorized objects, and, finally, from categorized waste into numerical data. When collecting the samples, we learned to develop an understanding of the potential contents of the waste bags by observing and feeling them and assessing their weight. Furthermore, as we categorized the collected sample, in addition to looking and smelling, we learned to shake, poke and smush waste to identify it and to negotiate the categories, simultaneously accepting potential errors in the process of categorization, thus relying on and exploring the relations between our different senses in the knowledge production process (Vannini, 2024, p. 13). Finally, in the phase of weighing the waste, we learned to understand the big picture of the sorting study and paid less attention to single objects but more to the different signifying material qualities of the waste. We also learned to notice exceptions to the ‘typical’ composition of waste when weighing it and were thus able to make notes about these exceptions on the Excel spreadsheet.

Our analysis also illustrates the compromises, flaws, and contradictions that the practices of categorizing waste may entail. These issues relate mainly to the interplay between the senses and the sensory qualities of waste. In the first phase of translation, we highlighted how acquiring embodied ways of knowing waste did not always lead to more accurate knowledge production, but in some cases, quite the opposite. Gaining more knowledge on waste may, paradoxically, lead to a willingness to avoid and thus not know certain smells, sensations and bodily reactions. The second phase of translation illustrated how sensory labor is a limited form of knowledge production, as accurate categorization of waste would in some cases require more information about the origins of certain waste objects. Further, the need to wear protective gear, and the constant coping with strong sensory sensations (or sheer disgust) also hindered the possibilities of accurately categorizing waste. In the third and final phase of translation, these compromises in the practices of categorizing waste were set aside through weighing and documenting—a practice that turned the messy sensory labor of categorization into neat numbers. Weighing and documenting formed a specific kind of technique of un/knowing (Alexander & O’Hare, 2023) that made both the sensory qualities of waste and the flaws of the sensory labor invisible. As a whole, our analysis thus illustrates the epistemological and calculative (Alexander & O’Hare, 2023) techniques of knowing stuff that you would not necessarily want to know about.

While some studies in feminist STS have illustrated how the objects and subjects of science are always in the making (Barad, 2003; Haraway, 1989), our analysis shows how the interplay between the senses and sensory qualities of waste may shape this making. Waste scholars have discussed, for example, the visceral registers of living with waste (Hawkins, 2006; Hawkins & Muecke, 2003) and the sensorial practices of knowing waste (Kinnunen, 2021), but these studies have not specifically explored how sensory experiences shape scientific knowledge production on waste. Waste has a capability to affect our bodies through senses, and related to this, the constant coping that dealing with waste requires shapes both the practices of categorization and knowledge production processes. The sensory qualities of waste did not affect us only when doing the actual work of sorting. We could sense the effects of this sensory labor even after the sorting sessions, as heavy fatigue and numbing of senses, and the lingering of rotten smell in our noses. The constant sensorial coping with the challenging stuff was both physically and mentally demanding.

Our study also has wider contributions to the understanding of waste policies in the context of the circular economy. The specific difficulties that relate to creating statistical knowledge on waste show that knowing waste is not only about management, calculation, and efficient processes, as often framed in the ethos of the circular economy (see Holmberg & Ideland, 2021) but also about less neat and sterile acts, such as looking, shaking, lifting, smelling, and poking. Moreover, the problems that relate to the interplay between senses and waste do not concern only statistical knowledge production for the purposes of waste management, but also household sorting practices that are crucial for the operation of the circular economy. While waste is sorted on a much smaller scale in households, sorting practices still demand everyday sensorial work from consumers and a close connection with waste on an everyday basis. As the ability of waste to evoke strong sensory experiences may hinder the possibilities of sorting waste accurately in the context of the waste composition study, so it may also hinder accurate sorting practices in households. People may not want to, for example, open mouldy jars that have been buried in the fridges for years, to wash them to sort them correctly, but rather put them in mixed waste to avoid disgusting smells and visual sensations. Dividing waste into separate streams rather than one ‘chunk’ makes one’s relationship with waste complex and multifaceted. Waste cannot just be quickly discarded into a bin; one must look at it closely, touch it and, overall, spend some time with it. These dimensions of practicing circularity in one’s everyday life are often overlooked in the technocratic mainstream discourse on waste, recycling and circular economy (see e.g. Hobson et al., 2021; Korsunova et al., 2021; Wheeler & Glucksmann, 2013).

Making the sensory labor of producing knowledge on waste (with all its flaws and contradictions) invisible through the calculative techniques of measuring and documenting is a question of framing ‘credible’ knowledge (Wynne, 1993). To make statistical knowledge on waste publicly acceptable and usable for the purposes of the circular economy policies, the practices of measuring and categorizing waste must be made to look flawless. However, the Finnish Environment Institute’s report on waste composition studies still acknowledges possible sources of error in the composition analysis process, stemming from impurities and humidity in mixed waste (which affects the accuracy of weighing procedures), the temporal and spatial fluxes in waste composition, problems in sampling and sorting, large waste objects and so on (Karppinen et al., 2021, pp. 23–24). It also acknowledges the ‘subjectivity’ of waste composition analysis practices, which can cause issues when comparing results (Karppinen et al., 2021, p. 26). Still the role of sensory labor remains mostly invisible in the final reports of waste composition analyses, and its role is also invisible in policies, programmes and public discussion. We argue that creating more sustainable and consistent circular economy policies requires taking sensory labor (and its potential ambivalences) seriously as a form of producing knowledge and economic value in different cultural, political and economic contexts.
